# Idiopathic Ovarian Vein Thrombosis: A Rare Cause of Abdominal Pain

**DOI:** 10.7759/cureus.16756

**Published:** 2021-07-30

**Authors:** Nayha Tahir, Robin Sherchan, Aneeba Farooqi, Jishna Shrestha, Hafiz Muhammad Jeelani

**Affiliations:** 1 Internal Medicine, Northwestern Medicine McHenry Hospital, Rosalind Franklin University of Medicine and Science, McHenry, USA

**Keywords:** idiopathic ovarian vein thrombosis, hypercoagulable state, septic pelvic thrombophlebitis, vitamin k antagonists, direct-acting oral anticoagulants

## Abstract

Ovarian vein thrombosis (OVT) is a rare entity. It is usually seen in hypercoagulable states such as pregnancy, peripartum period, active malignancy, recent pelvic surgeries, pelvic infections, and inherited or acquired thrombophilias. Idiopathic OVT is exceedingly rare. We report a case of OVT in a healthy 42-year-old post-menopausal female presenting with right lower quadrant abdominal pain for four days. The patient denied any recent pelvic surgery, pelvic infection, or any family history of thrombophilia. Right ovarian vein thrombosis was found on a computed tomography scan of the abdomen and pelvis. Laboratory workup including hypercoagulability studies was normal. The patient was treated with a therapeutic dose of lower molecular weight heparin and later transitioned to rivaroxaban for three to six months. This case emphasizes OVT as a differential diagnosis of lower abdominal pain in healthy females. Currently, there are no standard guidelines for the duration of anticoagulation in OVT, however based on literature review, deep venous thrombosis treatment guidelines can be followed.

## Introduction

Ovarian vein thrombosis (OVT) is commonly seen in hypercoagulable states. OVT in the postpartum period has been reported in approximately less than 0.19% of pregnancies [1]. Other etiologies of OVT are inherited or acquired thrombophilias and idiopathic OVT. Idiopathic OVT is extremely rare and has been so far documented in only 4 to 16% of cases of OVT [1]. Common symptoms associated with OVT include lower abdomen pain, adnexal mass, fever, and gastrointestinal symptoms. Early diagnosis and prompt treatment can prevent life-threatening complications like sepsis and pulmonary embolism.

## Case presentation

**Figure 1:**
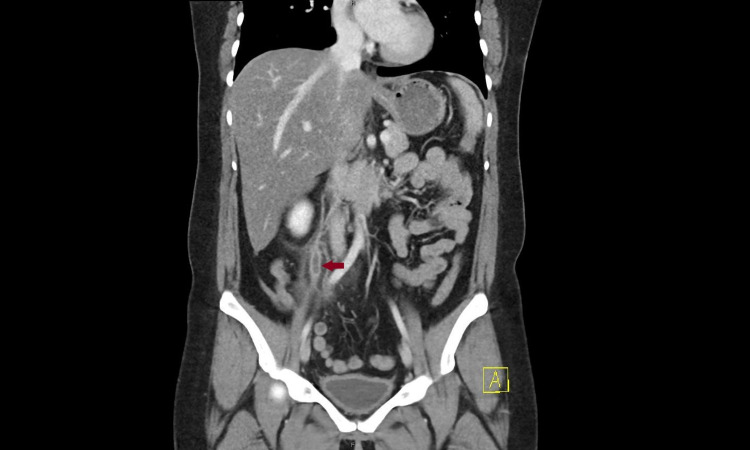
Coronal view of computed tomography scan with contrast of abdomen and pelvis showing right ovarian vein thrombosis (Arrow)

**Figure 2:**
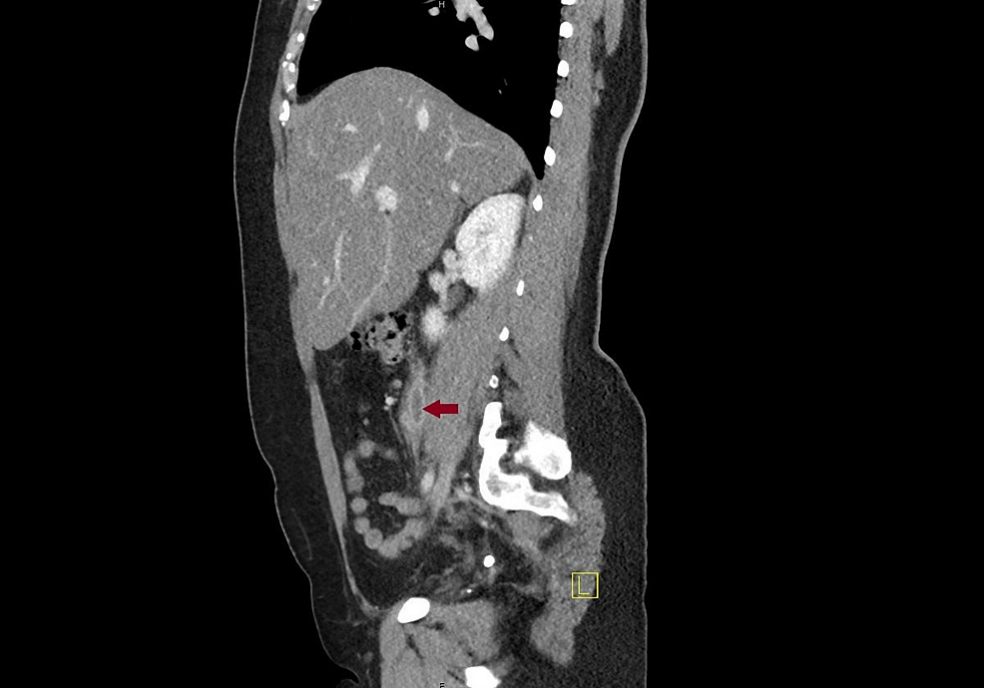
Sagittal view of computed tomography scan with contrast of abdomen and pelvis showing right ovarian vein thrombosis (Arrow)

**Figure 3:**
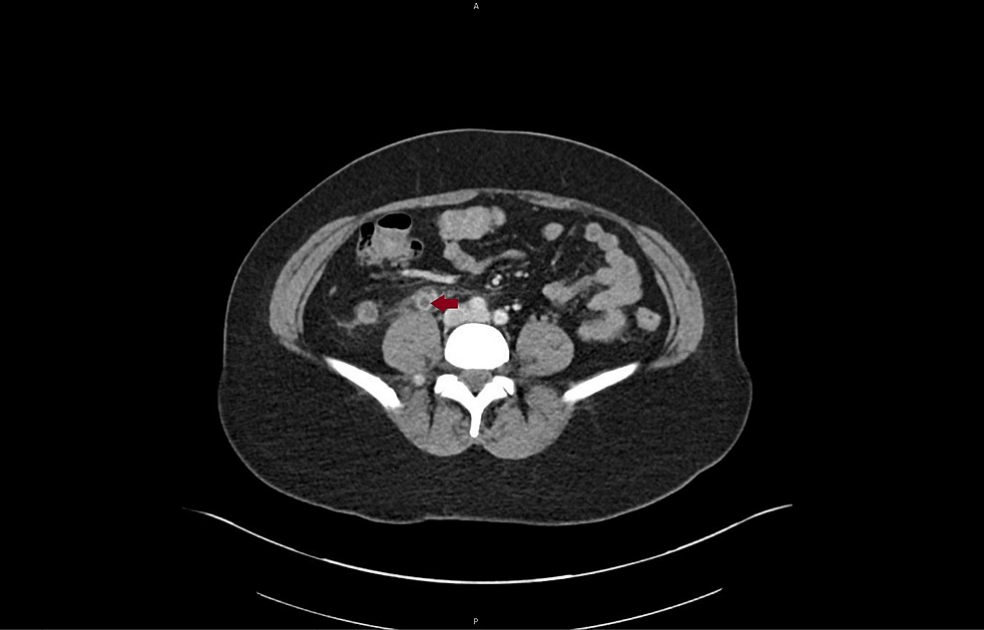
Axial section of computed tomography scan with contrast of abdomen and pelvis showing right ovarian vein thrombosis (Arrow)

A 42-year-old female presented to the emergency department with abdominal pain for four days. Past medical history was pertinent for total abdominal hysterectomy with bilateral salpingo-oophorectomy for stage 1b serous cystic ovarian neoplasm diagnosed eight years ago with the patient being in full remission. The pain was severe in intensity, throbbing, sharp, with radiation to the right thigh, aggravated with food, and with no other associated symptoms like fever, chills, nausea, vomiting, chest pain, leg swelling, leg pain, lightheadedness, or dizziness. She also denied any recent immobility, trauma, and infection. Vitals were stable on presentation and the physical exam was significant for right lower quadrant tenderness. Lab workup was unrevealing except for mild elevation in alanine aminotransferase (ALT) and aspartate aminotransferase (AST). Human chorionic gonadotropin (hCG) was negative. Computed Tomography (CT) with the contrast of abdomen and pelvis showed right ovarian vein thrombosis as seen in Figure 1, Figure 2, and Figure 3. 

Other pertinent negative tests included B2 glycoprotein, Factor V Leiden mutation, Protein C and S activity, Prothrombin mutation, Plasminogen Activator Inhibitor-1 (PAI-1), CA-125, Janus Kinase 2 (JAK2) mutation, anticardiolipin antibodies, and dilute russell viper venom time (DRVVT) confirmation test. The patient was initially started with a therapeutic dose of low molecular weight heparin (LMWH) with relief of symptoms the next day and was later transitioned to rivaroxaban upon discharge.

## Discussion

OVT occurs in the setting of Virchow’s triad, which includes venous stasis, coagulation cascade activation and venous damage leading to hypercoagulable state, hence more common in pregnancy, postpartum, malignancy, and thrombophilias [2-4]. Less frequently, pelvic infections can also cause OVT and should be considered among differentials [1]. OVT is a rare type of venous thrombosis [5] with idiopathic ovarian vein thrombosis being an even more rare entity [1]. Cases of OVT are typically life-threatening when occurring in the postpartum setting, however, in patients who underwent total abdominal hysterectomy with bilateral salpingo-oophorectomy like our patients, cases are typically asymptomatic. Moreover, asymptomatic unilateral OVT has been shown to occur in 80% of patients on routine computed tomography (CT) scan at three to 20 months after undergoing this procedure for cancer. Interestingly, 75% of cases were right sided, similar to our patient [6]. In contrast to reported cases, our patient had a symptomatic presentation of OVT occurring more than eight years after total abdominal hysterectomy with salpingo-oophorectomy. Symptoms of OVT are usually related to clot deposition and can range from a mild lower quadrant localized abdominal pain to severe excruciating pain with pelvic radiation, flank pain, or dyspnea if presenting as pulmonary embolism [7]. Typically the initial diagnostic study used in suspected cases is duplex doppler ultrasound, however it has been shown only to have a 50% detection rate. CT scan of the pelvis should be considered as it has a superior sensitivity at 77% and specificity at 62%. The most accurate but also most costly diagnostic test would be magnetic resonance angiography (MRA) which has a sensitivity and specificity of 100% in OVT detection [8].

Anticoagulation is the cornerstone of OVT treatment [1]. The recommended duration of therapy for women with OVT is three to six months [9,10]. In patients with cancer, incidentally detected isolated OVT can be monitored without anticoagulation [6]. Direct oral anticoagulants (DOACs) have not been specifically studied in patients with OVT however some case series and reports are present where patients with OVTs have been treated with direct oral anticoagulants (DOACs) with similar efficacy [11,12]. If there is a suspicion for concomitant infection, then broad-spectrum antibiotics are crucial [1,13]. DOACs are contraindicated during lactation as they are excreted into breast milk. Vitamin K antagonists (VKAs) can be used during lactation. Low molecular weight heparin remains the drug of choice in the antenatal period and cancer-related OVT [1,14,15]. Thrombolytic drugs (alteplase, urokinase) have been used in few cases but are associated with a high risk of bleeding therefore they should be reserved for patients with massive thrombosis. Retrievable inferior vena cava (IVC) filters can be used in cases of active bleeding including emergent surgical cases where anticoagulation is contraindicated. Surgical treatment with ovarian vein excision or ligation is rarely performed nowadays [1]. OVT may extend to the left renal veins in case of the left ovarian vein thrombosis or the IVC in cases of right ovarian vein thrombosis. Incidence of PE is less common in OVT than in lower extremity deep vein thrombosis (DVT) [1,16]. OVT can also lead to pelvic congestion syndrome and recurrent VTE [7]. OVT associated with septic pelvic thrombophlebitis could evolve into septic shock or emboli, which have a high mortality rate. 

With the use of antibiotics and anticoagulants, mortality rate has decreased [17]. Physicians should keep in mind this rare diagnosis when evaluating female patients with lower abdominal pain. More research is warranted in regards to anticoagulant agents and timing of therapy in the management of symptomatic and asymptomatic OVT. 

## Conclusions

OVT should be considered among differentials of lower abdominal pain in women. The CT scan of the abdomen and pelvis with contrast is a reasonable imaging to start the work up for OVT. The current recommendations for treatment are to follow guidelines for lower extremity DVT. The diagnosis of OVT requires a high level of suspicion, thorough history, physical exam, management by anticoagulants, and/or antibiotics if infection is suspected.
